# Patterns of observer error in scoring macromorphoscopic traits for population affinity

**DOI:** 10.1111/1556-4029.70063

**Published:** 2025-05-07

**Authors:** Leandi Liebenberg, Kyra E. Stull, Ericka N. L'Abbé

**Affiliations:** ^1^ Forensic Anthropology Research Centre, University of Pretoria Pretoria South Africa; ^2^ Department of Anthropology University of Nevada, Reno Reno Nevada USA

**Keywords:** ancestry, Cohen's kappa, cranium, forensic anthropology, observer experience, repeatability

## Abstract

Revising methodologies is essential to understand the limitations and biases inherent in certain methods, which is crucial for obtaining reliable results. Due to the subjective nature of non‐metric methods, variation in trait scoring and its impact on accurately classifying biological parameters remains a concern that requires further investigation. This study aimed to examine the effects of observer experience, familiarity with the method, and different statistical approaches on the repeatability of macromorphoscopic traits in the cranium for population affinity. Seventeen traits were scored on a sample of 10 crania by five observers with varying experience levels. Intra‐observer agreement ranged from moderate to perfect, with three traits—inferior nasal margin, nasal bone shape, and nasal overgrowth demonstrating—the lowest agreement. Overall, inter‐observer repeatability ranged from poor to substantial agreement. After a group discussion on the scoring procedure and subsequent rescoring of the crania, a slight improvement in agreement was observed, with kappa values shifting towards moderate and substantial levels. Each observer exhibited variation in the repeatability of different traits. While general experience did not consistently translate into proficiency with the method, familiarity with the specific traits and scoring procedures contributed to more consistent results. Therefore, method‐specific training is crucial before applying the MMS traits in practice. Additionally, the choice of statistical approaches—such as applying different weights to Cohen's kappa based on data type—can influence the perceived reliability of a method. Practitioners should select weights and tests that are most appropriate for the data type of each trait being analyzed.

**Highlights**

- Method‐specific training is more impactful than general experience in scoring MMS traits.
- Interpersonal preferences affect trait repeatability, with no clear trend for problematic traits.
- Appropriate weights for ordinal versus nominal traits are needed to best gauge the degree of discrepancy.
- Trait repeatability may be impacted by standards not capturing all variation across populations.
- A universal threshold for kappa repeatability is needed to ensure consistency in forensic practice.

## 1 INTRODUCTION

While validation tests gauge the external validity of the performance of a method, reliability studies measure the repeatability or consistency of variables used in that method. Studies evaluating methodological approaches provide insights into their potential limitations and biases, which is crucial to obtaining reliable results. The subjective nature of non‐metric techniques, coupled with trait score variation and its presumed implications on the accurate classification of biological parameters, remains an enduring issue that requires further study [1, 2]. This continuous concern has prompted extensive research, with numerous studies dedicated to identifying and quantifying sources of observer error in diverse topics such as bone pathology, age estimation, and the classification of sex and population affinity [2, 3, 4, 5, 6].

In terms of non‐metric methods for population affinity, Hefner [1] introduced a robust methodology that utilized line drawings and definitions to be used to visually assess and score each trait, along with software designed to facilitate data collection. Over time, the method has been further refined to include a greater number of traits, more precise definitions, and a photographic atlas [7, 8]. With this scientifically acceptable framework in place, subsequent researchers also began to further explore what is now termed macromorphoscopic (MMS) traits. For example, Klales and Kenyhercz [9] conducted a validation study to evaluate the reliability of the amended method. While their findings support the external validity of the MMS approach, some points of concern and areas for improvement were also noted. One of their key conclusions was the necessity for training in the use of the traits before any practical application. Observer experience remains one of the most significant contributors to discrepancies in morphoscopic trait analysis [2, 4]. While published figures and descriptions are designed in such a way as to theoretically enable anyone to score the traits, this ideal is not consistently achieved [2]. Validation studies often report poorer results compared to the original publications. One potential explanation for this is that initial studies typically involve the developer of the method, either through direct data collection or indirectly through training in how to use the method [2, 4]. As such, the reported results may inadvertently underestimate scoring challenges that only become apparent in independent studies. Indeed, reproducibility testing is essential, but results from independent researchers give a more realistic reflection of the method as it would be used in practice.

Past research has demonstrated that individuals with greater experience working with skeletal remains tend to achieve more consistent results, irrespective of their familiarity with a specific method [2, 4, 5]. This consistency has been attributed to experienced practitioners having been exposed to a greater range of human variation, enabling them to identify and recognize subtle skeletal differences more effectively compared to their less experienced peers. However, whether general experience translates into proficiency in scoring may depend on the particular method being used. For instance, Klales et al. [2] observed that more experienced practitioners yielded more reliable scores for traits of the cranium and pubic bone used for sex estimation [10, 11]. Yet, while less experienced observers displayed greater trait score variability, the results still exhibited good overall agreement. This suggests that knowledge on skeletal variation may contribute more to the application of these methods than formal training on the specific scoring systems [2]. In contrast, when assessing MMS traits to estimate population affinity, Klales and Kenyhercz [9] showed that experienced practitioners were indeed more consistent; however, the agreement for most scores was only slight to moderate, indicating the need for method‐specific training prior to application. Kamnikar et al. [5] support this conclusion, noting that long‐term familiarity with the use of the MMS method reduces the likelihood of extreme trait scores, reinforcing the value of continued method‐specific experience in achieving reliable results.

The need for prior training in MMS traits may stem from the larger number of traits and the more variable and complex scoring system compared to methods used for sex estimation. For sex estimation, techniques like the Walker [10] method assess five cranial traits, while the Klales et al. [11] method evaluates three traits on the pubic bone. Both methods make use of a consistent ordinal scale (scores ranging from 1 to 5), with values representing an increasing gradation in trait expression. Conversely, the MMS traits for population affinity initially encompassed 11 traits [1], but have been expanded to include 17 traits in more recent publications [7, 8]. The scoring approaches for MMS traits vary widely: some traits (such as anterior nasal spine) are scored ordinally with ranked values; others (like orbit shape) are nominal, categorizing traits without inherent ranking. Additionally, some traits are scored dichotomously, where the trait is either absent or present (like the post‐bregmatic depression) [7, 8]. This variation in trait codification introduces greater complexity, which may increase trait discrepancies, particularly for practitioners without prior training or familiarity with the traits or their underlying data types.

The specific methodologies employed in validation studies can also significantly influence score variability, thereby complicating comparisons across studies. Factors such as sample characteristics and the number of trials or observers can impact the outcomes. For example, smaller sample sizes may lack representation of specific traits or trait states, potentially skewing results. A sample devoid of any crania with nasal overgrowth might exhibit perfect agreement for that trait, yet it does not necessarily accurately indicate an observer's ability to score or identify the trait in a broader context. Statistical choices and their application can further influence results. In biological anthropology, Cohen's kappa is a widely used measure of observer agreement. However, variations in kappa methodology, such as whether to apply weighting to traits, are not uniformly agreed upon. A weighted kappa may be better suited to ordinal traits, as it considers the severity of score disagreements and allows larger discrepancies to be penalized more heavily [5, 12]. Ultimately, it is not recommended to compare kappa values across variables with different prevalence or bias, or traits that are measured on different scales [13]. Despite these insights, the impact of methodological choices and kappa values on anthropological analyses remains underexplored [2]. For MMS traits, many studies have made use of the traditionally employed unweighted kappa calculations [e.g., 1, 9, 14], while more recent studies have argued for the use of a weighted Cohen's kappa [e.g. 5, 15, 16]. However, limited comparative research exists to evaluate how these statistical variations influence observer agreement outcomes [6].

The aim of this paper was to investigate sources of variation in trait scores when assessing MMS traits on the cranium for population affinity. This involved examining factors such as observer experience, familiarity with the method, and the influence of different statistical approaches on the repeatability of the method.

## 2 MATERIALS AND METHODS

The sample consisted of 10 crania selected from the Pretoria Bone Collection, in the Department of Anatomy, at the University of Pretoria. Ethical approval (770/2018) to conduct the study was obtained from the Faculty of Health Sciences Research Ethics Committee at the University of Pretoria. To ensure a wide variety of trait expressions, thus avoiding statistical issues with trait prevalence, the sample included black and white South African males and females. The demographic information was not disclosed to the observers to prevent any potential cognitive bias. The 17 MMS traits (Table 1) were scored on each cranium by five different observers. The observers differed in their levels of experience regarding osteology and forensic anthropology as well as their experience with the traits. Table 2 provides a summary of the observers, with information on their level of education and number of years they have been involved with forensic casework, data collection, and osteological research at the time the data were collected. Observer A (the principal investigator) has extensive experience in the field, having worked with skeletal material for 10 years, which includes forensic case analysis, data collection, and teaching. Additionally, they also have extensive experience with the traits, having received training (from an expert with experience using the traits, but not a method developer) and self‐trained with the figures and descriptions for c. 3 years prior to data collection. Given this extensive background, observer A was designated as the reference standard for evaluating consistency across all other observers. Observer B is the only other participant familiar with some of the traits, having published on the subject. Observers C–E had no experience with the traits and vary in their general experience.

**TABLE 1:** MMS traits.

Anterior nasal spine	ANS	Nasofrontal suture	NFS
Inferior nasal aperture	INA	Orbital shape	OS
Interorbital breadth	IOB	Post‐bregmatic depression	PBD
Malar tubercle	MT	Posterior zygomatic tubercle	PZT
Nasal aperture shape	NAS	Supranasal suture	SPS
Nasal aperture width	NAW	Transverse palatine suture	TPS
Nasal bone contour	NBC	Palate shape	PS
Nasal bone shape	NBS	Zygomaticomaxillary suture	ZS
Nasal overgrowth	NO		

**TABLE 2:** Summary of observer experience.

Observer	Highest education/Employment	Trait experience	Osteology experience
A	PhD student, practicing forensic anthropologist	Extensive (scored the traits in >100 individuals, received training)	10 years' experience working with forensic cases and data collection in SA
B	PhD, practicing forensic anthropologist	Moderate (scored the traits in <100 individuals)	20 years' experience working with forensic cases and data collection in SA and USA
C	PhD student, practicing forensic anthropologist	Novice (has never scored the traits or used the method)	10 years' experience working with forensic cases and data collection in SA
D	MSc student	Novice (has never scored the traits or used the method)	2 years' experience working with forensic cases and data collection in SA
E	BSc undergraduate student	Novice (has never scored the traits or used the method)	Very limited experience working with skeletal material

The MMS traits were scored following the descriptions by Plemons and Hefner [7] as used in the “Macromorphoscopics Software” data collection module (*MMS* version 1.6.1) [17]. For the scoring, each observer was supplied with the *MMS* software and the *MMS* user guide. As per the recommendation of the MMS user manual, a contour gauge was provided to assist with the scoring of the nasal bone contour and the post‐bregmatic depression, and a clear ruler was provided to assist with the scoring of the malar tubercle and the posterior zygomatic tubercle. Only the left side was scored in the case of bilateral traits in this context to ensure the same variables were being scored, even though some variables are determined by greatest expression (e.g., posterior zygomatic tubercle).

Each of the five observers scored the crania by themselves, without discussing the scores with one another. After all observers had completed the scoring, a group discussion was held to deliberate on the scoring procedure. During this discussion, the observers went through the descriptions for each trait and how they each went about assigning a score and resolving scores for any traits they were conflicted about. A series of additional crania (independent of the ones being scored for analysis) were brought to the discussion session to showcase different examples of the traits as well as some variants that may complicate scoring. Each observer then rescored the same crania that were originally scored within a period of 4–6 weeks after the first round of scores. Once again, the observers scored the crania individually without discussing their scores. All the same tools (*MMS* software, user manual, contour gauge, and clear ruler) were made available to the observers.

The observer agreement was then assessed with Cohen's kappa, which was calculated with the *irr* package in R [18, 19]. The kappa coefficient measures the agreement between observers in assigning categorical variables adjusted by the standard measure of reliability that could be expected due to chance. Calculated kappa values can range from −1 to 1, where values closer to 1 indicate greater agreement. On the other hand, a negative value indicates agreement due to chance. There is currently no universally accepted cut‐off point for satisfactory observer agreement. However, to be consistent with nomenclature when describing the strength of agreement associated with kappa statistics, the parameters proposed by Landis and Koch [20] were used (Table 3).

**TABLE 3:** Description of Cohen's kappa agreement as described by Landis and Koch [20].

<0.00	Poor
0.00–0.20	Slight
0.21–0.40	Fair
0.41–0.60	Moderate
0.61–0.80	Substantial
0.81–1.00	Almost perfect

Different weights can be assigned to categorical variables depending on the data structure of the trait (i.e., binary, nominal, or ranked ordinal) and how harshly disagreement in a score should be penalized [12, 13]. While an unweighted kappa is suitable for binary and nominal structured traits (where any score disagreement is equally penalized), a weighted kappa should be considered for ordinal traits that have a specific rank or order to the scores [13]. To better explore the implications of different modifications to the statistical test, a series of analyses were run using different weights for the traits for the intra‐observer agreement. This included the traditional unweighted Cohen's kappa for all traits; linear‐weighted Cohen's kappa for all traits; and quadratic‐weighted Cohen's kappa for all traits. Lastly, a mix of unweighted (for binary and nominal traits) and quadratic‐weighted (for ordinal ranked traits) Cohen's kappa was applied to the appropriate traits. For the inter‐observer agreement, a mixed Cohen's kappa was selected to compare the scores for each additional observer with observer A to explore individual trends. A mean kappa value was then calculated from the pairwise comparisons for each trait to see the overall repeatability of the traits when considering all observers simultaneously. A Holm's adjustment was applied to avoid familywise error with multiple comparisons. While Fleiss' kappa is a common metric to assess agreement among multiple observers, its statistical power decreases with smaller sample sizes, particularly when analyzing data that tends to produce only moderate or low agreement (such as the inter‐observer agreement of MMS traits). Furthermore, Fleiss' kappa does not identify specific sources of disagreement among observers, limiting its utility for detailed reliability analysis. For these reasons, it was not utilized in the present study. The inter‐observer agreement was calculated both before and after the group discussion to see if more familiarity with the traits and the scoring procedure influenced the agreement.

## 3 RESULTS

The intra‐observer agreement was assessed using Cohen's kappa with varying weights assigned to the traits (Table 4). The mean kappa value varies depending on which weights are applied, with the unweighted kappa producing the lowest mean values, and the quadratic‐weighted kappa producing the highest mean values. The application of quadratic weights to the ordinally ranked traits (anterior nasal spine—ANS, inferior nasal margin—INA, malar tubercle—MT, nasal aperture width—NAW, and posterior zygomatic tubercle—PZT) consistently yielded higher agreement scores than if no weights were assigned. Closer inspection of the raw data revealed that this is because the scores for the ordinally ranked traits were nearly always within one score. The binary traits (nasal overgrowth—NO, post‐bregmatic depression—PBD) yielded the same kappa values regardless of weighting, as there are only two possible scores that can be assigned. Unranked traits with a greater number of trait states may yield scores that exhibit greater separation from the original score, often resulting in lower agreement values (i.e., an overestimation of error) if weights are assigned to them. Thus, using the correct weights that best suit each of the different traits based on their data structure is highly recommended as it gives the most realistic results.

**TABLE 4:** Comparison of intra‐observer agreement using Cohen's kappa with different weights.

	Unweighted kappa	Linear‐weighted kappa	Quadratic‐weighted kappa	Trait‐specific mixed weights
ANS	0.62	0.72	0.82	0.82
INA	**0.47**	**0.52**	0.78	**0.47**
IOB	0.70	0.76	0.83	0.83
MT	**0.43**	**0.57**	0.72	0.72
NAS	0.62	0.62	0.62	0.62
NAW	0.84	0.87	0.91	0.91
NBC	0.64	0.75	0.84	0.64
NBS	**0.43**	0.63	0.79	**0.43**
NO	**0.41**	**0.41**	**0.41**	**0.41**
NFS	0.83	0.72	0.62	0.83
OS	0.80	0.84	0.89	0.80
PBD	0.74	0.74	0.74	0.74
PZT	**0.41**	**0.55**	0.69	0.69
SPS	0.81	0.72	0.64	0.81
TPS	1.00	1.00	1.00	1.00
PS	0.71	0.63	**0.56**	0.71
ZS	0.74	0.74	0.76	0.74
Mean	0.66	0.69	0.74	0.72
Min	0.41	0.41	0.41	0.41
Max	1.00	1.00	1.00	1.00

*Note*: Bold indicates values with moderate agreement or lower (<0.60).

With the appropriate weights assigned to each trait, the intra‐observer agreement ranged from 0.41 (moderate) to 1.00 (perfect), with nasal overgrowth (NO) and transverse palatine suture (TPS) performing the worst and best, respectively.

The inter‐observer repeatability of the traits was compared among five observers with varying experience. This was done by comparing each observer to observer A, and then calculating the mean kappa value for each trait (Table 5). Overall, the mean kappa values ranged between −0.13 (poor) and 0.66 (substantial), with the nasal bone contour (NBC) performing the worst and interorbital breadth (IOB) performing the best. Interorbital breadth was the only trait to demonstrate substantial agreement, with all other traits showing moderate to poor repeatability. The performance of each observer compared to observer A revealed variable results. For example, the anterior nasal spine (ANS) showed fair agreement between observers A and B (0.29) but showed almost perfect agreement between observers A and D (0.82). Conversely, orbit shape (OS) showed almost perfect agreement between observers A and B (0.83), while there was only slight agreement between observers A and D (0.15). Thus, each observer varied in which traits they were less/more repeatable.

**TABLE 5:** Inter‐observer agreement using Cohen's kappa among multiple observers.

	Obs A – Obs B	Obs A – Obs C	Obs A – Obs D	Obs A – Obs E	Mean
ANS	0.29	0.42	**0.82**	**0.67**	0.55
INA	0.08	0.49	0.11	0.36	0.26
IOB	0.58	**0.74**	**0.91**	0.42	**0.66**
MT	0.55	**0.69**	0.55	0.35	0.53
NAS	0.48	−0.15	0.51	0.36	0.30
NAW	**0.66**	0.55	0.30	0.40	0.48
NBC	−0.09	−0.23	NaN	−0.09	−0.13
NBS	0.39	0.30	0.38	0.46	0.38
NO	0.05	−0.11	0.29	−0.11	0.03
NFS	0.40	**0.65**	0.47	0.41	0.48
OS	**0.83**	0.43	0.15	0.43	0.46
PBD	0.21	−0.32	NaN	0.05	−0.02
PZT	0.48	0.31	0.53	0.49	0.45
SPS	0.03	−0.08	0.34	0.61	0.23
TPS	0.57	0.21	0.29	0.09	0.29
PS	NaN	−0.33	0.43	0.37	0.16
ZS	**0.73**	0.52	0.52	**0.62**	0.60
Mean	0.39	0.24	0.44	0.35	0.34
Min	−0.09	−0.33	0.11	−0.11	−0.13
Max	0.83	0.74	0.91	0.67	0.66

*Note*: Scores recorded before any trait discussion. Bold indicates substantial agreement or higher (>0.61).

In some instances, kappa values could not be calculated (e.g., “NaN” was obtained for nasal bone contour—NBC, post‐bregmatic depression—PBD, and palate shape—PS). NaN (“Not a Number”) appears in R when calculating Cohen's kappa if there is no variation in the ratings (e.g., one observer assigns the same score to all items) or if there is perfect disagreement between raters. In both cases, the denominator in the kappa formula becomes zero, rendering the calculation undefined. An undefined kappa indicates potential prevalence issues with the sample, such that only one trait state is present in the sample and is being scored the most (in other words there is little to no variation in the ratings). However, as it only happened sporadically and did not happen with the intra‐observer tests, it likely indicates that one observer in the pairwise comparisons was assigning the same score to all of the crania for the traits in question, while other observer pairs were assigning more variable scores; i.e., a bias issue (from the observer) rather than a prevalence issue (from the sample).

All the observers rescored the same crania following a group discussion on the scoring procedure (Table 6). Overall, the mean kappa values increased after the discussion, ranging from −0.04 (poor) to 0.75 (substantial), with the supranasal suture (SPS) performing the worst and nasal aperture width (NAW) performing the best. Five traits demonstrated substantial agreement values or higher (anterior nasal spine—ANS, interorbital breadth—IOB, nasal aperture width—NAW, nasal overgrowth—NO, and posterior zygomatic tubercle—PZT) compared to the first round of scores, where only one trait demonstrated substantial agreement. Notably, four of the five traits with substantial agreement are ordinally ranked.

**TABLE 6:** Inter‐observer agreement using Cohen's kappa among multiple observers.

	Obs A – Obs B	Obs A – Obs C	Obs A – Obs D	Obs A – Obs E	Mean
ANS	0.44	**0.66**	**1.00**	**0.64**	**0.69**
INA	0.23	**0.86**	−0.11	0.45	0.36
IOB	**0.77**	**0.91**	0.31	**0.77**	**0.69**
MT	0.44	0.59	0.59	0.58	0.55
NAS	**0.83**	0.24	0.41	−0.06	0.36
NAW	**0.81**	**0.91**	0.58	**0.72**	**0.75**
NBC	0.21	0.13	0.25	0.05	0.16
NBS	−0.06	0.44	0.55	0.26	0.30
NO	**0.80**	**0.78**	0.60	0.60	**0.70**
NFS	0.33	**0.67**	0.49	0.53	0.51
OS	0.39	0.57	**0.80**	0.09	0.46
PBD	−0.11	0.29	−0.15	**1.00**	0.26
PZT	0.21	**0.72**	**0.88**	**0.72**	**0.64**
SPS	0.17	0.11	−0.32	−0.11	−0.04
TPS	0.10	0.47	0.18	0.37	0.28
PS	**0.74**	0.18	0.55	0.28	0.44
ZS	0.11	**1.00**	0.06	0.33	0.37
Mean	0.38	0.56	0.39	0.42	0.44
Min	−0.11	0.11	−0.11	−0.11	−0.04
Max	0.83	0.91	1.00	0.77	0.75

*Note*: Scores recorded after the discussion session. Bold indicates substantial agreement or higher (>0.61).

Mixed results were observed when comparing the mean kappa values for each observer. Even though observer B has five traits with substantial agreement, they presented with the overall lowest mean, indicating more variation in their scores. The mean kappa values decreased for both observers B and D after the discussion. For observer B, the agreement remained fair, while with observer D, the overall agreement dropped from moderate to fair. Both observers C and E showed increased agreement from fair to moderate after the trait discussion, with observer C demonstrating the most marked increase. Table S1 presents the intra‐observer agreement for each additional observer to demonstrate how their scores changed after the discussion session.

## 4 DISCUSSION

Non‐metric methods, by their very nature, tend to be subjective and are often susceptible to variability and bias [1, 21]. This study aimed to explore the sources of trait score variation when assessing the MMS traits of the cranium for population affinity, shedding light on factors that could affect consistency and accuracy. Prior research has highlighted the critical roles of statistical approaches, observer expertise, method‐specific training, and inherent population differences in shaping the reliability of morphological trait scoring [2, 5, 9]. By delving deeper into these influences, this study offers a clearer understanding of the challenges and nuances involved in applying non‐metric methods effectively.

Firstly, each statistical method used to compare reliability has its own set of assumptions and limitations, which can lead to conflicting outcomes when the assumptions are met or violated. Klales et al. [2] highlight the challenges in evaluating results of reliability studies for sex estimation, particularly as researchers employ diverse statistical measures for observer agreement, ranging from Cohen's kappa with varying weights, to intraclass correlations. While most studies typically rely on Cohen's kappa to evaluate the reliability of the MMS traits, a lack of consensus exists on the differential weighting of traits. The current study revealed significant variability in agreement rates depending on the weight assigned to the traits. It is important that practitioners be cognizant of the fact that MMS traits, although comparable to scoring systems such as those of Walker [10] or Klales et al. [11], differ in data structure and the number of trait expressions. As such, a universally suitable kappa weight is not feasible for all traits. The choice of statistical test must account for these structural differences in the data, both when comparing methods and interpreting results across multiple studies. For traits that are ordinally ranked with a logical order, the quadratic‐weighted kappa offers a realistic measure of agreement. Since ordinal scores are quasi‐continuous with overlapping boundaries, small differences in scores (such as being within one score or trait expression) should not be penalized as severely as larger discrepancies (e.g., two or more scores out). Among some of the most frequently cited studies, including those by Hefner [1], L'Abbé et al. [14], and Klales and Kenyhercz [9], there was no indication whether weights were applied to the traits, suggesting that unweighted kappa was likely used. This approach may underestimate agreement for ordinal traits. Conversely, Maier [15] and Kamnikar et al. [5] employed quadratic weights for all traits, which while appropriate for the ordinal traits, can overstate agreement for nominal traits. As nominal traits lack gradational overlap, it requires a different approach, as disagreement in scoring reflects an error in identifying particular shapes or variants rather than misjudging the relative size of a skeletal feature. Thus, applying quadratic weights to all traits can result in underestimation of error for nominal traits. Table 7 presents the performance of each trait across various studies. However, making direct comparisons and reaching definitive conclusions remains difficult due to potential variability in the parameters used to calculate Cohen's kappa in each study. Ultimately, different weights can influence the apparent reliability of a method, and practitioners should apply weights and tests that are suitable to the data type being analyzed for each trait.

**TABLE 7:** Comparison of a selection of published inter‐ and intra‐observer agreement rates in scoring the MMS traits.

Intra‐observer							Inter‐observer					
	Current study	Hefner [1]	L'abbé et al. [14]	Maier [15]	Kamnikar et al. [5]	Corron et al. [6]	Current study^a^	Hefner [1]	L'abbé et al. [14]	Klales and Kenyhercz [9]^a^	Klales and Kenyhercz [9]^b^	Corron et al. [6]
ANS	0.821	0.422	0.81	0.759	0.49	0.7	0.685	0.506	0.55	0.165	−0.250	0.80
INA	0.468	0.964	0.58	–	0.63	0.833	0.357	0.376	0.65	0.284	−0.522	0.75
IOB	0.833	0.857	0.53	0.666	0.64	0.615	0.689	0.325	0.44	0.412	0.242	0.60
MT	0.717	0.929	0.53	0.658	0.10	0.64	0.551	0.470	0.44	0.382	−0.538	0.73
NAS	0.615	–	–	–	–	0.526	0.356	–	–	0.324	0.412	0.83
NAW	0.906	0.929	0.68	0.702	0.64	0.308	0.752	0.732	0.56	0.167	0.167	0.57
NBC	0.643	0.810	0.74	0.624	0.69	0.231	0.159	0.231	0.54	0.141	0.032	0.87
NBS	0.429	–	–	–	–	0.69	0.297	–	–	0.198	0.155	0.66
NO	0.412	1.00	0.64	0.922	0.58	0.556	0.695	1.00	0.73	0.374	0.500	1.00
NFS	0.833	–	–	–	–	–	0.506	–	–	0.281	0.032	–
OS	0.804	–	–	–	–	1.00	0.464	–	–	0.453	0.375	1.00
PBD	0.737	0.820	–	0.768	0.62	–	0.255	0.232	–	0.411	−0.250	–
PZT	0.688	–	–	–	<0.01	0.766	0.635	–	–	0.251	0.365	0.71
SPS	0.808	0.468	–	0.634	–	–	−0.040	0.650	–	0.586	0.412	–
TPS	1.000	1.00	–	0.714	–	–	0.281	0.700	0.38	0.485	0.767	–
PS	0.714	–	–	0.610	–	1.00	0.437	–	–	–	–	0.83
ZS	0.737	0.857	0.39	0.600	0.06	–	0.374	0.541	0.11	0.166	0.357	–

^a^ Experienced observer.

^b^ Inexperienced observer.

Cohen's kappa is also associated with certain limitations, notably issues of prevalence and bias, often termed the paradoxes of the kappa statistic. Prevalence arises when one trait is disproportionately represented, making it difficult to discern true agreement beyond what might occur with chance [22, 23]. This imbalance can result from the sampling process. For instance, if a sample used for reliability testing primarily comprises groups in which the trait is absent or exceedingly rare, the agreement score might not accurately reflect an observer's ability to assess the trait correctly when it is present. On the other hand, bias refers to how often observers assign scores to specific categories; i.e., it pertains to individual interpretation of descriptive criteria or reference diagrams [22, 23]. While methodical sample selection can mitigate the effects of prevalence, controlling for bias is significantly more difficult. Bias issues can prevent the calculation of a reliable coefficient with Cohen's kappa, as was observed with the first round of inter‐observer scores prior to the group discussion. To address the paradoxes, the calculation of additional prevalence and bias indices has been recommended; however, the indices are only applicable to nominal data [22]. While such calculations are relatively easy for binary traits (scored as present or absent), it becomes increasingly complex for traits with multiple trait expressions. Another proposed solution is the prevalence and bias‐adjusted kappa (PABAK), but similarly applies only to nominal data [23]. The combination of several different data types within a single method further complicates the analysis and comparison of traits. No uniform approach is universally applicable, and practitioners need to demonstrate heightened awareness of strategies to circumvent potential issues pertaining to repeatability testing, especially when assessing non‐metric traits. This includes selecting sufficiently large samples, ensuring the inclusion of the widest possible range of traits, using repeatability measures suited to the data structure and number of states per trait, and providing sufficient detail to facilitate comparability of findings across studies.

Despite differences in the quantification of trait repeatability, results from the current study were compared to previously published research. The intra‐observer agreement is equivalent to rates from other published studies (see Table 7 for comparisons). Among the traits analyzed, three demonstrated moderate repeatability, which is the lowest agreement level observed in the current study; this includes inferior nasal margin (INA), nasal overgrowth (NO), and nasal bone shape (NBS). The inferior nasal margin, in particular, has one of the greatest numbers of trait expressions (with states ranging from 1 to 5). This trait assesses whether the floor of the nasal aperture is smooth or sloping as it transitions to the maxilla, or whether the aperture is demarcated by a ridge of bone [7, 8]. The change of the slope from one score to the next is gradual and quite difficult to discern from photographs [15]. While studies involving the developer of the method reported substantial to near‐perfect agreement [1, 5], few independent studies have published intra‐observer agreement rates. Notably, both South African studies – the current study and that of L'Abbé et al. [14] – recorded moderate agreement for the inferior nasal margin (INA). The lower agreement compared to Hefner [1] may stem from differing interpretations of the descriptions and images but could also reflect differences in trait expression attributable to population variation [5].

Both the nasal overgrowth (NO) and nasal bone shape (NBS) may exhibit lower repeatability due to subtle trait variations. Nasal overgrowth (NO) assesses the projection of the nasal bones beyond the maxilla and is scored as either present or absent. Generally, traits with only two sharply differentiated states are expected to have great reliability [24]. However, Merchant [16] has raised concerns regarding the definition of nasal overgrowth, particularly how variations, such as separation of the nasal bones from the maxilla, correspond with the description of true nasal overgrowth. Finally, nasal bone shape (NBS) evaluates the degree of “pinching” and “bulging” of the nasal bones. In this study, discrepancies were observed between the comparative drawings and the actual specimens. In many cases, the crania did not match the degree of nasal bone “bulging” depicted in the images, potentially indicating greater population variation than accounted for in the trait descriptions. Additionally, nasal bones were often noted to be asymmetrical. While Hefner and Linde [8] recommend using the most pronounced expression for asymmetrical traits in general, this guideline has not been explicitly applied to the nasal bone shape [15]. Thus, the discrepancies between observers may not only be due to observer experience but could also stem from the trait descriptions and line drawings themselves not adequately capturing the full range of expression found in the South African population. This line of research would benefit from directly comparing the current sample to the original reference sample to explore potential population differences and their effects on accurately scoring the traits across various global populations.

The inter‐observer agreement in this study was notably lower than the intra‐observer agreement, with several traits demonstrating poor repeatability. Ideally, the difference between inter‐ and intra‐observer agreement should be minimal, as large discrepancies suggest that while an observer can consistently score traits within their own evaluations, it does not guarantee the reliability of a method in accurately scoring traits among multiple individuals. Nevertheless, literature consistently reports much higher intra‐observer agreement for the MMS traits [1, 14], likely attributable to familiarity and experience with the method. The current study conducted a comparison based on levels of general experience, which revealed that less experienced individuals exhibited fewer traits with higher levels of agreement. However, this trend was not consistently reflected in the mean kappa values calculated per observer, as even the observers with substantial general experience showed comparably low repeatability. Collectively, none of the traits consistently exhibited poor repeatability across all observers. Instead, each observer presented with different traits that achieved the highest and lowest levels of repeatability, respectively. This variation likely stems from the diverse nature of the traits being assessed (e.g., size, shape, latent continuous traits, etc.), as observers may demonstrate preferences for certain trait types over others. For example, observer C showed greater proficiency with size‐related traits but struggled more with those focused on shape. These subjective, interpersonal preferences become intertwined with the overall repeatability and reliability of the method, highlighting the influence of individual biases on scoring consistency. The variability among observers suggests that in the absence of additional guidance or shared knowledge, each observer resorts to individualized scoring approaches for different traits. This assumption is substantiated by potential instances of scoring bias, as is evidenced by the numerous cases where it was not possible to calculate a kappa value for certain traits. Thus, general experience with skeletal material does not directly translate to competency in using the traits.

Numerous authors have emphasized the importance of method‐specific training to effectively use the MMS traits [5, 9]. While certainly not equivalent to continuous comprehensive training, a discussion session was held with all observers to explore whether even a modest degree of familiarity and additional instruction on the scoring procedure could improve reliability results. This session appeared to have a positive impact, with several traits demonstrating improved repeatability compared to previous scores. Although this improvement is once again not reflected in the mean kappa values for each observer. The discussion culminated in mixed results. Two observers (C and E) exhibited improved repeatability following the discussion, with their mean kappa values increasing from fair to moderate. Interestingly, these observers had varying levels of general experience, indicating that general experience alone does not contribute significantly to more consistent scoring of the traits. In contrast, the other two observers (B and D) demonstrated decreased repeatability compared to their initial scores. This result initially seemed unexpected, as observer B is the only other observer with prior experience with the traits, having previously published on the subject. However, their experience pertained to the traits as described in the original publication [1]. Since then, several modifications have been made, including the addition of more traits, adjustments to the trait scales, and the introduction of the MMS user interface, all of which may affect the intra‐observer agreement. Additionally, there has been limited research quantifying trait repeatability over extended periods, which could also contribute to lower agreement, even among those with prior experience using the traits [5].

In the second round of scoring, no instances of “NaN” values (i.e., cases where kappa values were undefined) were observed, suggesting that the discussion session may have helped to reduce potential scoring bias. While such discussions are certainly not a substitute for thorough method‐specific training, they play a valuable role in refining standard procedures, clarifying terminology, and addressing issues related to language, translation, and personal interpretation. These factors can significantly impact the consistency of non‐metric trait assessments [4]. Moreover, such discussions offer valuable insights into the strategies practitioners employ to address challenges in trait assessment, which can lead to error or bias. This is particularly important where established guidelines to resolve specific variations are lacking.

In the current study, several personal approaches to scoring the traits became apparent. Certain observers (e.g., observer A) placed a significant emphasis on tactile examination to assess the size of some traits, such as the anterior nasal spine (ANS), nasal aperture width (NAW), and posterior zygomatic tubercle (PZT). The repeatability of the above traits improved when the other observers adopted this approach. The use of different tools to visually assess certain traits also varied among the observers. To improve trait repeatability, the most recent guidelines recommend the use of a contour gauge to better visualize the nasal bone contour, and a clear ruler is recommended to examine the size of the malar tubercle and posterior zygomatic tubercle [5, 7, 8]. One observer commented on using the ruler to also assess the nasal aperture width and interorbital breadth, which essentially converts the trait to a measurement. Merchant [16] addresses ambiguity regarding the location and placement of the ruler to assess the posterior zygomatic (PZT) and malar tubercles (MT), ultimately highlighting a lack of consensus among their cohort of observers. While the exact placement of the ruler did not form part of the collective discussion in the current study, this omission is likely attributed to the observers using it infrequently. Throughout the training period, observer A attempted scoring both with and without the ruler and observed greater consistency when it was not used. Similarly, the contour gauge received limited preference to score nasal bone contour (NBC), and it was not used during data collection. In the group discussion, several observers noted that scoring the trait with the contour gauge consistently yielded the same score (despite the nasal region itself looking different), leading to repeatability poorer than chance and introducing bias in the scores. Following additional instructions provided by observer A, subsequent attempts to score nasal bone contour (NBC) without the contour gauge demonstrated improved repeatability; however, the kappa value remained quite low. The results support findings in the literature calling for training prior to using the traits in research or skeletal analyses [5, 9].

Although the importance of training cannot be overstated, Wilczak et al. [4] raise concerns regarding the potential implications of “second‐hand” and self‐training in scoring. Typically, developers of new methods offer training at workshops or through collaborative projects. However, as methods become more established and widely applied, the availability of training opportunities diminishes. As such, practitioners often need to rely on published descriptions and photographs, or training provided by independent individuals with some experience in the method. Although observers without direct training from developers can still produce consistent results (especially for intra‐observer agreement), the possibility exists that discrepancies may arise compared to the developers or other experts in the field [4]. Such discrepancies can lead to variations in trait frequencies between studies and ultimately decreased classification accuracy [2, 25]. Furthermore, discrepancies can become standard practice as they are passed down from one generation to the next through educational pedagogy [26]. Additional research needs to evaluate the precision and reliability of scoring the MMS traits, especially for the sake of data sharing and the collation of a global database. Ultimately, the observer agreement achieved in this research is in line with previous studies evaluating the MMS traits (Table 7), which have considered the repeatability satisfactory to justify its use in practice. Nevertheless, it is important to acknowledge that there is no established threshold for what constitutes an acceptable kappa value. Further deliberation is necessary to establish criteria for adequate levels of validity and reliability by which a method can be assessed for its applicability in forensic casework [26]. For the method to be a viable option to conduct population affinity estimation in South Africa, the forensic anthropology community responsible for assessing skeletal remains should be subjected to rigorous training in scoring the MMS traits prior to the method being used in analyses.

Online resources have the potential to enhance consistency in trait scoring on a global scale and help address discrepancies in a standardized manner. In a comprehensive study examining the qualitative assessment of pathological lesions on bone, Wilczak et al. [4] recommended several steps to potentially improve confidence and validity in scoring methods. These recommendations included fostering ongoing discussions on terminology and method refinement, making exemplar cases and case studies of trait variations available, and incorporating 3D models into the training and scoring procedure [4]. While Hefner and Linde [8] have published a photographic atlas showcasing the different MMS traits, challenges related to scoring the traits from 2D photographs have been discussed [16]. Other authors have also highlighted issues with scoring from 2D photographs, such as poor lighting, sub‐optimal angles in photographs, and the skeletal feature not being entirely in the plane of focus [4, 16, 27, 28]. This makes scoring particularly challenging for individuals that are relying on photographs for training purposes.

The use of 3D models is a potential solution for the challenges associated with 2D photographs. With technological advancements, forensic anthropologists are increasingly integrating 3D virtual reconstructions of bones into their research, educational practices, and forensic evidence reconstruction for legal testimony [28, 29]. In virtual 3D models, users can manipulate the image by rotating the bone or zooming in on specific features. The viewing software also allows for the application of different settings, such as varied lighting or textures, to enhance specific traits or features. In particular, 3D models derived from the Michigan State Forensic Anthropology Laboratory Donated Collection are available for both cranial and postcranial MMS traits via the 3D MMS initiative and offer a useful resource for comparative and educational purposes [30]. Incorporating 3D models into teaching has proven effective in improving comprehension and consistency among students in identifying skeletal features [28]. While 3D models offer several advantages, they are not considered a complete replacement for physical bones, particularly for traits that require palpation. Kuzminsky et al. [31] noted that although virtual models were suitable for experienced individuals in analyzing sex from the cranium, they prove less reliable for individuals with less experience. Thus, a combined approach utilizing real bones, 3D printed bones or casts alongside virtual models may yield optimal training. The impact of using 3D models (both virtual and physical) as part of MMS trait training should be further explored.

## 5 CONCLUSION

This study highlights the inherent challenges of using morphoscopic traits in skeletal analysis, particularly in ensuring consistency and reliability across observers. While statistical tests such as Cohen's kappa are widely used to assess agreement, some limitations necessitate careful consideration of weighting and data structure, particularly for nominal and ordinal traits. Prevalence and bias issues further complicate the interpretation of kappa values, reinforcing the need for methodical sample selection and awareness of scoring biases. The results from the current study align with previous research, demonstrating higher intra‐observer than inter‐observer agreement, with specific traits exhibiting lower repeatability due to subtle morphological variations. Importantly, general experience with skeletal material does not guarantee accurate trait assessment, emphasizing the necessity of method‐specific training. Discussion sessions resulted in improved agreement for some observers, suggesting that even minimal additional instruction can positively influence trait scoring. However, discussions cannot replace the level of training required to achieve reliable results that offer practical application for forensic skeletal analyses.

To improve reliability in using the MMS traits, future efforts should focus on standardizing training protocols, incorporating technological advancements such as 3D models, and considering the potential implication of population‐specific variation on observer agreement. These tools could enhance consistency in scoring and facilitate more accurate data collection across global forensic and anthropological contexts. Clearer criteria for acceptable agreement thresholds need to be considered and discussed in the discipline. Continued research and collaboration among practitioners will be key to refining these methods and improving their applicability in diverse populations. Ultimately, method refinement and validation should be an ongoing, globally collaborative effort.

## CONFLICT OF INTEREST STATEMENT

The authors have no competing interests to declare that are relevant to the content of this study.

## Supporting information


Table S1

## Data Availability

The dataset generated/analyzed during the current study is available from the corresponding author on reasonable request.
