# Supplementary material for: Patterns of observer error in scoring macromorphoscopic traits for population affinity
Source: J Forensic Sci. 2025 May 7;70(4):1489–500. doi: 10.1111/1556-4029.70063 (PMC12223330; doi:10.1111/1556-4029.70063)
Supplement: Supplementary file 1 — Table S1 [file JFO-70-1489-s001.docx]

| TABLE S1 Intra-observer agreement using Cohen’s kappa comparing the scores of the additional observers before and after discussion. Bold indicates substantial agreement or higher (>0.61). | | | | |
| --- | --- | --- | --- | --- |
|  | **Observer B** | **Observer C** | **Observer D** | **Observer E** |
| **ANS** | 0.35 | **0.61** | **1.00** | **0.73** |
| **INA** | 0.55 | 0.26 | 0.05 | 0.58 |
| **IOB** | **0.84** | **0.80** | 0.47 | 0.57 |
| **MT** | **0.69** | **0.94** | 0.09 | 0.29 |
| **NAS** | **0.68** | 0.29 | -0.17 | 0.19 |
| **NAW** | **0.73** | 0.55 | **0.65** | 0.36 |
| **NBC** | 0.28 | 0.15 | -0.08 | 0.44 |
| **NBS** | **0.70** | 0.43 | **0.71** | **0.69** |
| **NO** | 0.55 | 0.18 | 0.44 | 0.41 |
| **NFS** | 0.39 | 0.28 | **0.64** | -0.02 |
| **OS** | **0.67** | 0.57 | 0.49 | 0.47 |
| **PBD** | **0.62** | 0.09 | 0.00 | -0.18 |
| **PZT** | 0.13 | 0.00 | 0.49 | 0.25 |
| **SPS** | 0.10 | 0.40 | -0.11 | -0.03 |
| **TPS** | 0.18 | 0.55 | 0.50 | 0.52 |
| **PS** | 0.43 | 0.10 | 0.23 | 0.18 |
| **ZS** | 0.12 | 0.21 | 0.39 | **0.80** |
| ***Mean*** |  |  |  |  |
| ***Min*** |  |  |  |  |
| ***Max*** |  |  |  |  |
